# Prognostic value of serum CYFRA21-1 and CEA for non-small-cell lung cancer

**DOI:** 10.1002/cam4.493

**Published:** 2015-09-02

**Authors:** Zhi-Hui Zhang, Yun-Wei Han, Hui Liang, Le-Min Wang

**Affiliations:** 1 Department of Thoracic Surgery, Affiliated Hospital of Tai’an Medical UniversityTai’an, Shandong, 271000, China; 2 Department of Oncology, The First Affiliated Hospital of Sichuan Medical UniversityNo. 25 Taiping Street, Luzhou, Sichuan, 646000, China; 3 Department of Medical Imaging, The General Hospital of Jinan Military CommandJinan, Shandong, 250031, China; 4 Department of Thoracic Cardiovascular Surgery, Tongji Hospital, Tongji University School of MedicineShanghai, 200065, China

**Keywords:** Cancer, carcinoembryonic antigen, keratins, meta-analysis, non-small-cell lung cancer, prognosis

## Abstract

The aim of the study was to assess the clinical prognostic value of serum cytokeratin 19 fragment (CYFRA21-1) and carcinoembryonic antigen (CEA) for non-small-cell lung cancer (NSCLC) patients. Literatures related to effects of serum CYFRA21-1 and CEA on the prognosis of lung cancer patients were retrieved from databases such as PubMed, Springer Link, Embase, Wanfang, and CNKI. Meta-analysis was carried out using RevMan 5.1 software. Ten literatures involving 1990 NSCLC patients were selected in this study. Total survive estimation merging hazard ratio (HR) in all NSCLC patients with high-level serum CYFRA21-1 was 1.64 (95% CI 1.46–1.84, *P* < 0.001) and that in all NSCLC patients with high level serum CEA was 1.46 (95% CI 1.28–1.65, *P* < 0.001). Serum CYFRA21-1 and CEA can be used as prognostic factors of NSCLC patients. Combinative detection of the two indices will be more reliable.

## Introduction

Lung cancer is one of the most frequent malignant tumor and its mortality has been in first place among urban population died of malignant tumors 1. 85% of lung cancer are non-small cell lung cancer (NSCLC). Although previous studies have demonstrated that postoperative chemotherapy plays a significant role in extending life span of NSCLC patients 2,3, the 5-year survival is still up to 30–50% 4. Tumor makers of lung cancer, including Cytokeratin 19 fragment (CYFRA21-1), carcinoembryonic antigen (CEA), specific neurons enolase, CA-125, and squamous cell carcinoma antigen (SCC-Ag) 5 can be used for therapy detection and prognosis 6. CYFRA21-1 is a fragment of CK19 and a primitively expressed epithelial cytokeratin. Although complete cytokeratin molecular usually exists as in dissolvable form, there is solubility CYFRA21-1 in the serum of lung tumor patients. They are acidity (I type) subunit which may be released into serum after tumor metastasis and cytolysis and they are useful serum markers. NSCLC patients (including patients in prophase and advanced stage) with high-level serum CYFRA21-1 are poor prognosis. In other words, high serum CYFRA21-1 level may be a useful noninvasiveness marker to identify NSCLC risk. While it still need careful and abundant clinical studies to be confirmed 7. CEA is a glycoprotein in carcinoembryonic cell surface and is one of the earliest applied tumor markers to detect NSCLC. CEA increases in many tumor tissues such as lung cancer, gastrointestinal neoplasms, breast cancer, carcinoid, and liver cancer and become an independent prognosis factor for NSCLC 8. However, single-serum tumor marker is hard to reach ideal level in detecting sensitivity and specificity of lung cancer.

Among different tumor makers of NSCLC, CYFRA21-1 and CEA are widely studied. High serum CYFRA21-1 and CEA levels have similar negative effects on lung cancer prognosis. Previous studies indicated that the risk of mortality of NSCLC was associated with serum CYFRA21-1 and CEA levels 9–18. However, the conclusion remains controversial. This meta-analysis aims to investigate the relationship between NSCLC prognosis and serum CYFRA21-1 and CEA levels.

## Materials and Methods

### Literature retrieval

We retrieved literatures focusing on the effect of serum CYFRA21-1 and CEA on NSCLC prognosis in any level through searching databases such as PubMed, Springer Link, Embase, Wanfang, and CNKI (language was limited to English and Chinese). The terms were “CYFRA21-1” or “cytokeratin fragment 21-1” and “CEA” or “carcinoma embryonic antigen” and “NSCLC” or “Non-small cell lung cancer” or “lung adenocarcinoma” or “lung squamous” and “prognosis” and “survival.”

### Inclusion criteria and exclusion criteria

Inclusion criteria: (1) patients who were diagnosed as NSCLC by pathology; (2) initial distribution of the description was defined as operation treatment and other treatments such as chemotherapy, radiotherapy, the best supporting nursing care or the combination of all these methods; (3) serum CYFRA 21-1 levels were detected by ELISA method; (4) studies on the relationship between serum CYFRA21-1, CEA, and NSCLC prognosis in any levels; (5) all data were obtained from original literatures and only restricted at complete indices on NSCLC prognosis such as hazard ratio and 95% CI; (6) all data were analyzed by univariate and multivariate survival analysis; (7) full text can be obtained.

Exclusion criteria: (1) literatures which were repetitive report, with low quality and less information, congress abstract, severe bias, and impossible to be utilized; (2) literatures which only provided abstract but no full text information; (3) literatures which did not detect serum CYFRA21-1 and CEA levels.

### Data collection

The following data were collected: (1) gender; (2) age; (3) origin date (the day began to detect serum CYFRA21-1 and CEA); (4) classification based on metastasis tumor Tumor Node metastasis (TNM) standard procedure of the Union for International Cancer Control (UICC) 19; (5) histology subgroup 18; (6) serum CYFRA21-1 and CEA levels. All NSCLC were histologically classified into five groups, including adenocarcinoma, squamous cell carcinoma, adenosquamous carcinoma, bronchoalveolar carcinoma, and large cell carcinoma. Adenocarcinoma, bronchoalveolar carcinoma, and large cell carcinoma were all classified to other NSCLC since their low amount. Therefore, there were only three histology morphous to be analyzed.

### Statistical analysis

We calculated standard error (SE) according to the methods in literature from Parmar et al. 20 and regarding natural logarithm of Hazard Ratio (HR) and its SE were used to merge the HR. Since reference values of serum CYFRA21-1 and CEA internalized into literatures were different, we regarded those upper reference values as high-level serum CYFRA21-1 and CEA. We carried out homogeneity test for the internalized literatures and utilized fixed effects models to proceed weighted merger when *P* > 0.05, but random effects models was used if *P* < 0.05. The merged HR and its 95% CI were presented as forest plots. Taking SE of internalized HR natural logarithm value as abscissa and the HR natural logarithm value as ordinates, the funnel plot was drawn to describe publication bias. RevMan 5.1 statistics software (The Nordic Cochrane Centre, Cochrane Collaboration, Copenhagen) was utilized to carry out meta-analysis for all the data processing, regarding *P* < 0.05 as statistical significance.

## Results

### Literature selection and characteristics

**Figure 1:**
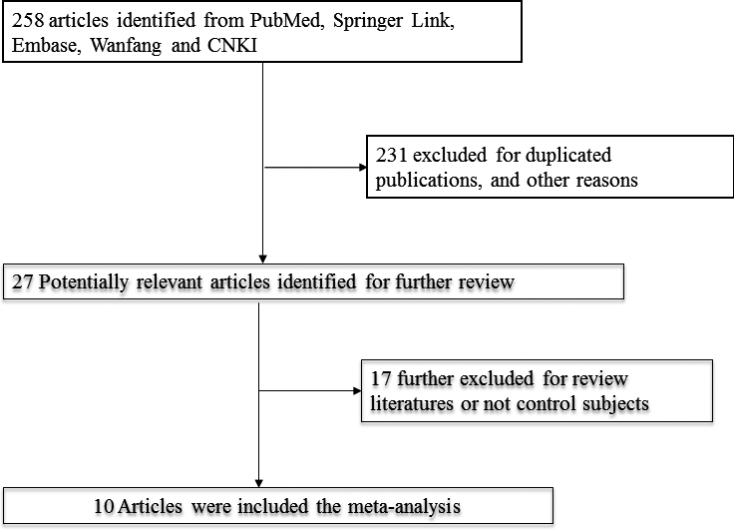
Flowchart of literatures identification.

As shown in Figure1, 258 literatures from databases such as PubMed, Springer Link, Embase, Wanfang, and CNKI were searched. Among them, 10 literatures 9–18 were selected according to inclusion criteria and exclusion criteria. The participants were from Czech Republic, Germany, Poland, Republic of Korea, Japan, China, Israel, and France. A total of 1990 patients with age ranging from 19 to 89 and follow-up ranging from 60 to 140 months were from the above countries and the therapies for them were recorded. All the 10 internalized literatures had studied many NSCLC prognosis-related factors such as serum CYFRA21-1, CEA levels, tissue type, pathology staging, differentiation level, age, sex, smoking, and SCC-Ag. Neuron-specific enolase (NSE), lactate dehydrogenase, white blood cell count and other biomarkers provided the results of univariate and multivariable analysis. This study selected HR in multivariable analysis to remove the mingle effects of other prognosis-related factors to serum CYFRA21-1 and CEA level and many NSCLC pathology types. The characteristics of included studies were shown in Table1.

**Table 1:** The characteristic of the included studies

Authors	Publication year	Country	Detection methods	Number of patients	Pathological stage	Univariate analysis	Multivariate analysis
Nisman et al.	1998	Israel	ELISA	94	I–IV	Yes	Yes
Barlési et al.	2004	France	ELISA	264	IIIB–IV	Yes	Yes
Kulpa et al.	2002	Poland	ELISA	200	I–IV	Yes	Yes
Matsuoka et al.	2007	Japan	ELISA	275	I	Yes	Yes
Tomita et al.	2010	Japan	ELISA	291	I–IV	Yes	Yes
Jung et al.	2011	Korea	ELISA	123	IIIB–IV	Yes	Yes
Muley et al.	2004	Germany	ELISA	153	I	Yes	Yes
Lin et al.	2012	China	ELISA	169	I–IV	Yes	Yes
Lee et al.	2012	Korea	ELISA	277	IV	Yes	Yes
Fiala et al.	2014	Czech Republic	ELISA	144	IIIB–IV	Yes	Yes

### Meta-analysis

**Figure 2:**
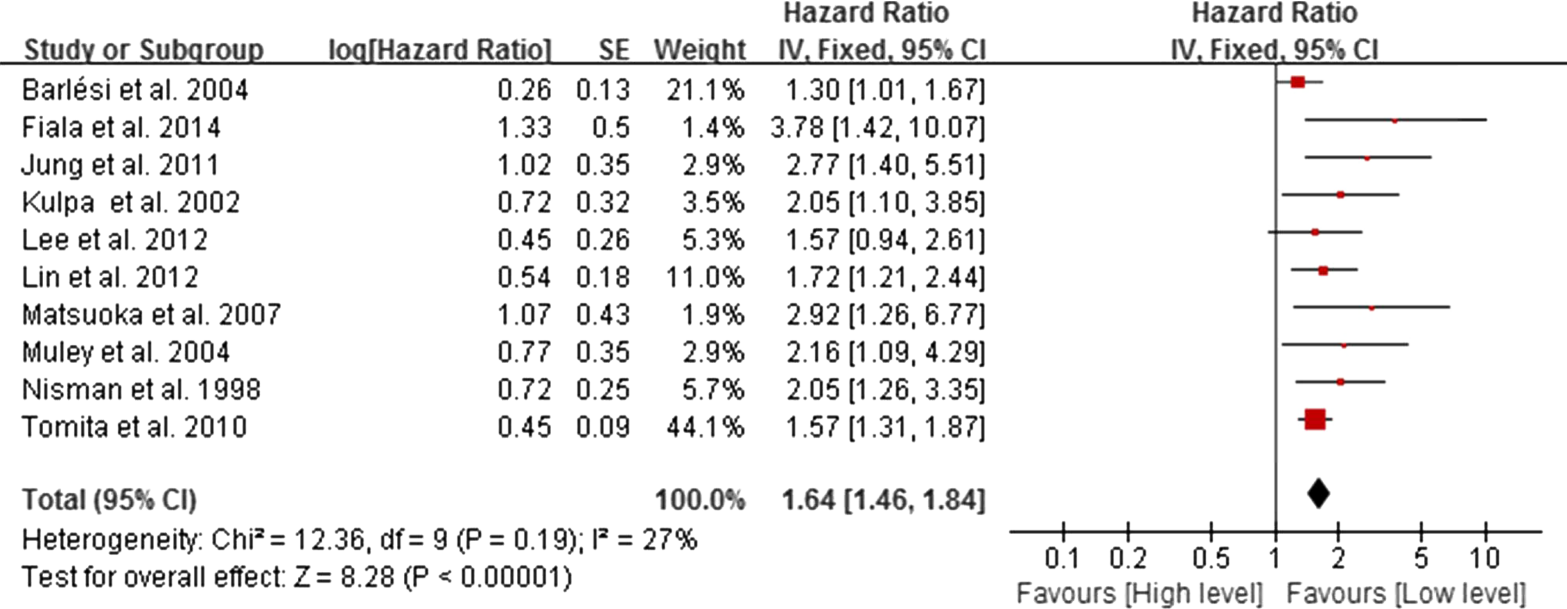
Forest plot of prognosis of NSCLC and CYFRA21-1 expression. The horizontal lines correspond to the study-specific HR and 95% CI, respectively. The area of the squares reflects the study-specific weight. The diamond represents the pooled results of HR and 95% CI. NSCLC, non-small-cell lung cancer; CYFRA21-1, cytokeratin 19 fragment.

**Figure 3:**
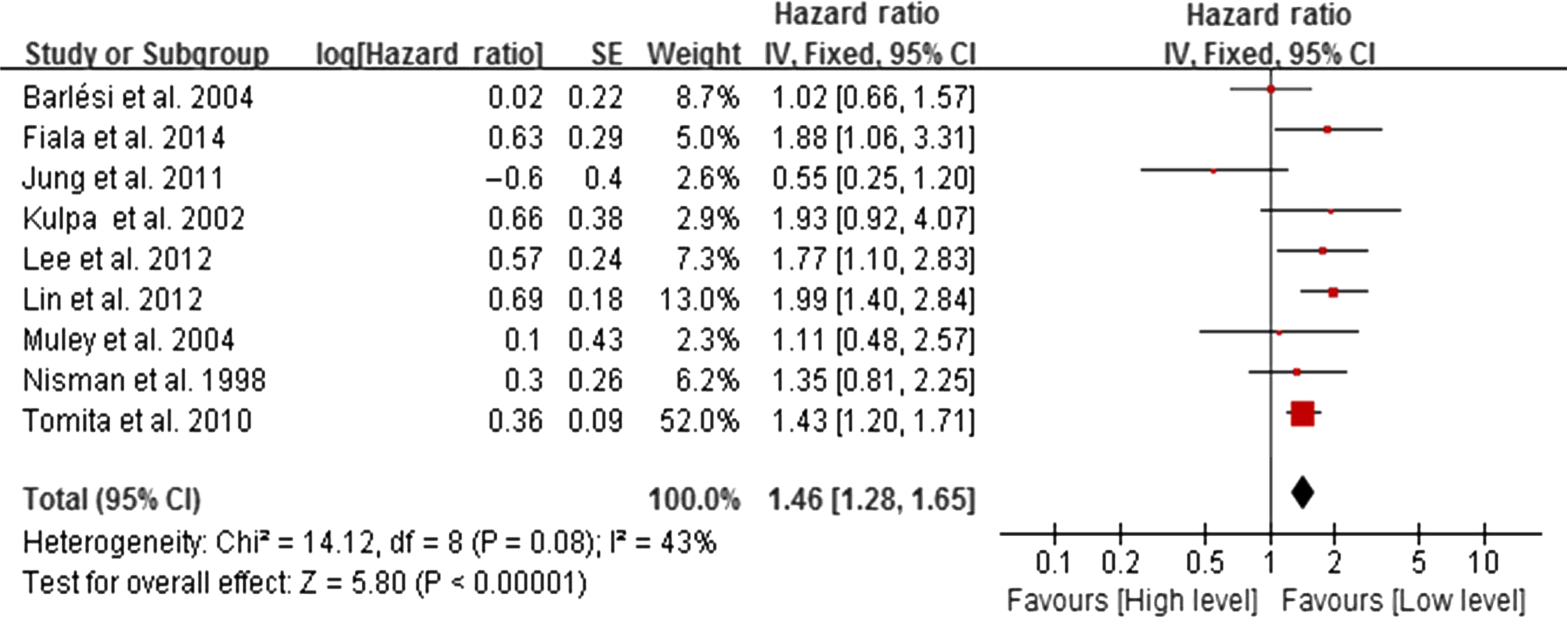
Forest plot of prognosis of NSCLC and CEA expression. The horizontal lines correspond to the study-specific HR and 95% CI, respectively. The area of the squares reflects the study-specific weight. The diamond represents the pooled results of HR and 95% CI. NSCLC, non-small-cell lung cancer; CEA, carcinoembryonic antigen.

We utilized fixed effect model because there was no significant heterogeneity across studies (*I*^2^ < 50%, *P* > 0.05). The HR in final meta-analysis was 1.64 (95% CI 1.46–1.84, *P* < 0.001, Fig.2). The HR in final meta-analysis was 1.46 (95% CI 1.28–1.65, *P* < 0.001, Fig.3).

### Publication bias analysis

**Figure 4:**
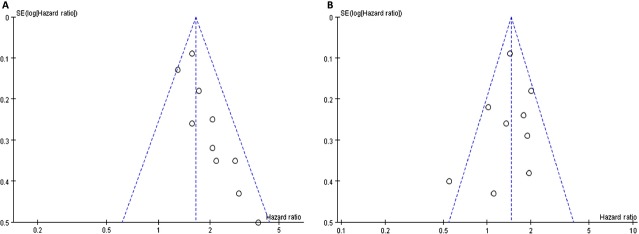
Funnel plot for publication bias test. Each point represents a separate study for the indicated association. Log HR represents the natural logarithm of HR. The vertical line represents the mean effects size. (A) CYFRA21-1; (B) CEA. CYFRA21-1, cytokeratin 19 fragment; CEA, carcinoembryonic antigen.

The funnel plot showed symmetry, which suggested there were no significant publication bias (Fig.4).

## Discussion

In the present study, we included 10 literatures in which the detection of CYFRA21-1 and CEA for NSCLC patients to perform a meta-analysis. The results showed that merged HR of NSCLC patients with high serum CYFRA21-1 level is 1.64, and with high serum CEA level is 1.46, suggesting that patients with high serum CYFRA21-1 level or high CEA level are poor prognosis.

The included 10 studies are from different institutes. Therefore, we think the further analysis for subgroup is questionable. However, each research center classified tumors according to the standard procedure on metastatic tumor TNM from UICC and the therapies (especially chemotherapy schedule), which provided the best support for the homogenicity of the study. In addition, each included research was carried out by multivariable analysis to eliminate the mingle effects of other prognosis-related factors. In this study, we utilized the median age (64 years) as threshold since age affects the life span of all the study individuals, which may lead to limitation in this study. Furthermore, we selected the studies published in Chinese and English, which may result in selection bias.

Sensitivity and specificity in NSCLC of single detection of serum CYFRA21-1 or CEA are not enough. Many scholars claimed that combination of detection of these two indices should improve the positive ratio of lung cancer diagnosis and evaluation of prognosis 21. Therefore, not a single research could determine the prognosis’ significance of tumor markers mentioned in their literatures. Most publication or individual propose that high serum CYFRA21-1 and CEA levels suggesting poor prognosis only based on the effect levels of the measure value to mortality and the reliability for NSCLC prognosis. Further study will determine how to integrate this information to decide the treatment protocols.

However, there were several limitations that must be taken into account when considering the above-mentioned results: on one hand, tumor depth and nodal status were risk factors for prognosis. However, we did not consider these risk factors in the present study. On the other hand, postoperative adjuvant chemotherapy has demonstrated a clear survival benefit compared to treatment with surgery alone. However, some included studies failed to provide such information, which might have affected the result.

In conclusion, in the present study, we have determined the significance of CYFRA21-1 and CEA levels to NSCLC poor prognosis. Further, large-sample-size cohort studies focusing on this issue are warranted.

## Conflict of Interest

None declared.
